# Supplementary figures and images for: The Influenza B Virus Victoria and Yamagata Lineages Display Distinct Cell Tropism and Infection-Induced Host Gene Expression in Human Nasal Epithelial Cell Cultures
Source: Viruses. 2023 Sep 20;15(9):1956. doi: 10.3390/v15091956 (PMC10537232; doi:10.3390/v15091956)

A)

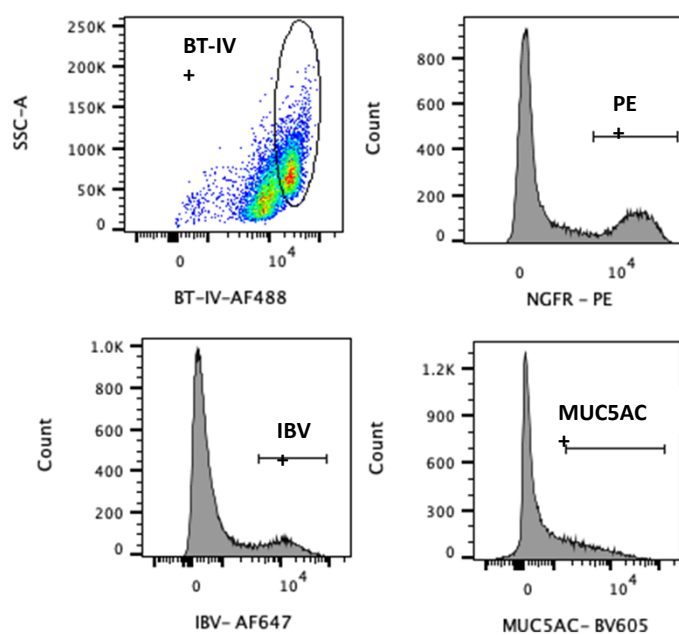

B)

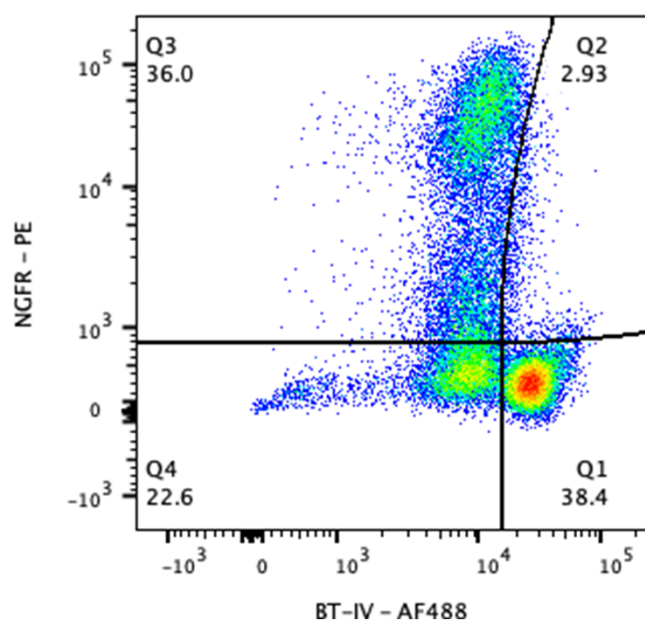

C)

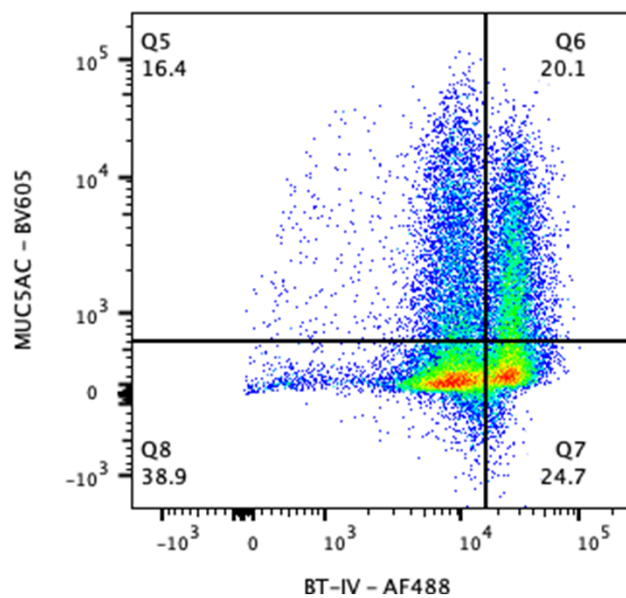

D)

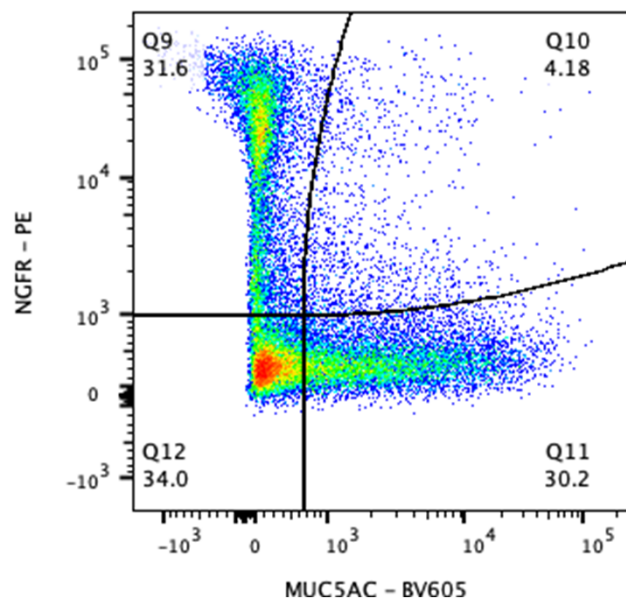

Supplement: Supplementary file 1 [file viruses-15-01956-s001.zip › Sup Figure 1.pdf]

A)

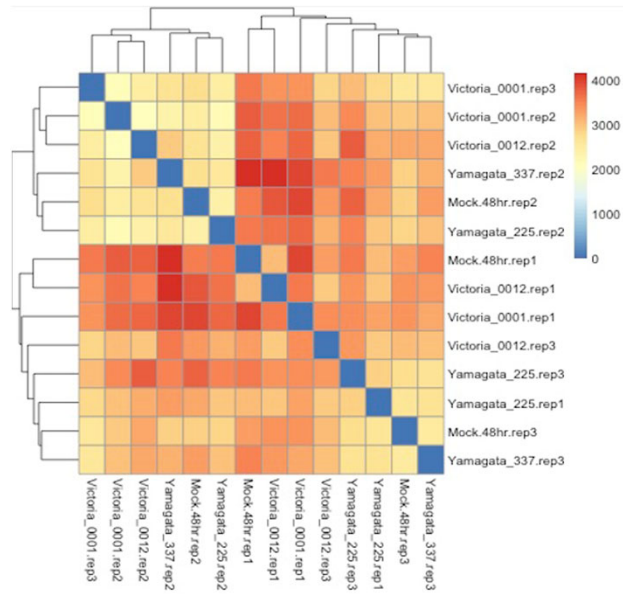

B)

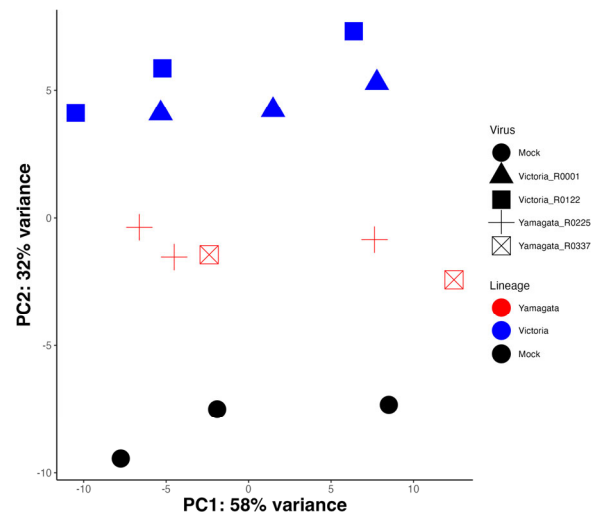

Supplement: Supplementary file 1 [file viruses-15-01956-s001.zip › Sup Figure 2.pdf]
